# 肺癌患者的肺康复治疗

**DOI:** 10.3779/j.issn.1009-3419.2011.09.09

**Published:** 2011-09-20

**Authors:** 艳洁 乔, 小明 邱, 清华 周

**Affiliations:** 300052 天津，天津市肺癌研究所，天津市肺癌转移与肿瘤微环境重点实验室，天津医科大学总医院肺部肿瘤外科 Tianjin Key Laboratory of Lung Cancer Metastasis and Tumor Microenvironment, Tianjin Lung Cancer Institute, Department of Lung Cancer Surgery, Tianjin Medical University General Hospital, Tianjin 300052, China

**Keywords:** 肺肿瘤, 康复治疗, Lung neoplasms, Rehabilitation

## Abstract

肺康复治疗是对有症状、日常生活能力下降的慢性呼吸系统疾病患者采取的一项有循证医学证据、多学科、全面干预的非药物治疗方法。肺康复治疗在肺癌中的应用还处于探索阶段，但初步的临床研究已显示它能有效地改善肺癌患者的运动耐量和生活质量、减少手术并发症并增加手术机会，具有重要的应用前景。

## Abstract (en)

Pulmonary rehabilitation is an evidence-based, multidisciplinary and comprehensive non-medication intervention for patients with chronic respiratory diseases who are symptomatic and often have decreased daily life activities. Applications of pulmonary rehabilitaion in the management of patients with lung cancer are only recently emerging. Preliminary studies showed its effects in improving exercise tolerance, health-related quality of life and surgical candidacy and decreasing surgical morbidity. It shows promise as a therapeutic intervention in the management of lung cancer.

肺癌是当今世界上对人类健康和生命威胁最大的恶性肿瘤，也是临床治疗疗效最差的恶性肿瘤。据估计，2008年全世界有160万人诊断为肺癌，占所有肿瘤的13%，约140万人死于肺癌，占所有肿瘤死亡的18%^[1]^。尽管近年来肺癌的化疗、放疗、靶向治疗以及免疫治疗取得了很大的进展，但外科治疗仍然是最有效的可能获得长期生存的治疗手段。而能否进行外科手术不仅取决于肿瘤部位和分期，还取决于患者的身体状况。研究^[2]^显示新诊断肺癌患者中，73%的男性和53%的女性合并有临床表现的慢性阻塞性肺病（chronic obstructive pulmonary disease, COPD），这些合并症可导致患者心肺功能损害，呼吸困难等症状加重，不仅使得手术风险增加，甚至使部分患者失去手术机会。肺癌手术本身引起的患者呼吸生理紊乱、肺组织容量减少、膈肌运动障碍及手术创伤等，必然导致术后呼吸循环功能损害，降低患者的生活质量^[3, 4]^。对于无法手术的肺癌患者，肿瘤本身会引起乏力、呼吸困难等症状，而放化疗等的副作用，会进一步加重这些症状。因此，在肺癌患者的整个治疗过程中，都有必要对改善心肺功能和提高生活质量采取针对性的治疗措施。

肺康复治疗是对有症状、日常生活能力下降的慢性呼吸系统疾病患者采取的一项有循证医学证据、多学科、全面干预的非药物治疗方法，旨在减轻慢性呼吸性疾病患者的呼吸困难，乏力等症状、提高运动耐力及生活质量、改善患者心理障碍及社会适应能力。全面的肺康复治疗包括：患者病情评估、运动训练、呼吸肌训练、教育及心理行为干预、氧疗和无创通气、营养治疗等，其中运动训练是综合性肺康复治疗的基石。研究^[5-8]^表明肺康复治疗能显著增加慢性阻塞性肺疾病患者接受肺减容和肺移植手术的机会，并明显提高患者运动耐力、减轻呼吸困难症状和改善生活质量。

由于肺康复治疗在慢性阻塞性肺疾病治疗中显示能明显改善心肺功能和生活质量，人们开始探索将其应用于肺癌患者的整个治疗过程中，近5年来开始有关于肺康复治疗对肺癌患者疗效的报道，本文就这些研究做一综述。

## 1 肺康复治疗的内容和评价

肺康复治疗在COPD患者的治疗中是一项被广泛认可的非药物治疗措施，其疗效和科学性已被大量临床试验证实，基于这些研究证据美国胸科医生协会（American College of Clinical Pharmacy, ACCP）和美国心肺康复协会（American Association of Cardiovascular and Pulmonary Rehabilitation, AACVPR）于1997年制定了循证医学肺康复指南，并于2007年对该指南进行了更新，对肺康复治疗进行了规范。综合的肺康复治疗方案包括对患者的评估、运动训练、宣传教育和社会心理支持等，体现了多学科合作、个体化治疗和关注身体及社会机能的特点，其中运动训练是基础和必须的^[7]^。

目前肺癌患者的肺康复治疗的内容主要是借鉴较成熟的COPD患者肺康复治疗的方案，以运动训练为主，还包括健康教育和营养支持。Andersen等^[9]^将COPD的肺康复治疗方案应用于肺癌患者，观察能否改善肺癌患者的身体状况和生活质量。研究入组了45例患者，肺康复治疗为期7周，每周2次。具体措施包括步行锻炼、处理呼吸困难的健康教育以及每日饮食指导等。结果显示肺癌患者接受肺康复治疗后能提高身体状况，治疗后增量往返步行测试增加了9%，耐力往返步行测试增加了109%，而肺功能和生活质量评分没有明显改善。然而其中过半患者未能完成治疗，而且身体状况有改善的患者很多也不能在家继续坚持锻炼，提示对于肺癌患者需要制定更加切实可行的治疗方案。

肺癌患者肺康复治疗的运动训练与COPD患者相似，内容包括：①下肢运动训练：如步行、蹬车、爬楼梯、游泳、跑步等，是肺康复治疗的关键性核心内容，能增强患者心肺运动功能和运动能力；②上肢运动训练：如两上肢绕圈、重复提举重物平肩等形式，上肢运动训练可增加前臂运动能力，减少通气需求；③呼吸肌训练：包括缩唇呼吸和腹式呼吸，临床上常用的还有吹气球练习等，可改善患者呼吸肌功能，减轻呼吸困难的症状^[7]^。

运动训练的强度标准：①有氧运动强度多采用心肺运动试验（cardiopulmonary exercise testing, CPET）评定，达到最大耗氧量（VO_2_max）20%-40%的运动量为低强度，60%-80%的运动量为高强度。COPD患者下肢高强度训练比低强度训练产生更大的生理学获益，且低强度和高强度训练均产生临床获益^[7]^；②耐力训练强度通常使用最大肌力（one-rep max, 1RM）的百分比表示，60%-70%的1RM为低强度，70%-80%的1RM为中强度，80%-100%的1RM为高强度。目前推荐的肺癌患者肺康复治疗的运动处方是从每周2天、每天10 min、中低强度的运动训练开始，逐步达到每周3天-5天、每天30 min、中高强度的运动训练^[10]^。

运动训练效果的评价方式：①心肺运动试验：包括功率自行车和平板运动试验，能全面客观地评价人体的最大有氧代谢能力和心肺储备能力，是评价运动训练效果的标准方法。采用评价指标分别有峰耗氧量（VO_2peak_）、最大耗氧量（VO_2_max）、最大公斤耗氧量（VO_2_max/kg）和代谢当量（metabolic equivalent of energy, MET）等。研究^[11]^发现VO_2peak_可作为肺癌手术并发症的独立预测指标和肺癌手术风险的评价指标，VO_2peak_ < 12 mL/kg/min与 > 20 mL/kg/min的患者相比，心肺并发症的发生率和死亡率分别是8倍和13倍，提示通过肺康复治疗提高VO_2peak_有可能降低手术并发症和改善预后；②6 min步行距离试验（six-minute walk distance, 6MWD）：以患者6 min内步行的最大距离为评价指标，该方法简单易行，重复性好，具有较好耐受性，更能反映日常活动能力。在COPD患者的研究^[12]^中发现，6MWD > 350 m的患者中65%的患者平均生存期为67个月，而6MWD < 350 m的患者中仅39%的患者平均生存期为67个月。但6MWD对肺癌手术风险的评估和预后判断尚无相关研究数据；③往返步行试验（shuttle walk test, SWT）：包括增量往返步行试验（incremental shuttle walk test, ISWT）和耐量往返步行试验（endurance shuttle walk test, ESWT），是在录音机指导下逐渐增加速度或以某一运动强度的速度在距离10 m的地方来回行走，所行走距离作为评价指标。

肺康复治疗的其它效果评价指标：①呼吸困难评价：常用Borg评分（10分制）来评价呼吸困难程度，分值越高表示呼吸困难程度越严重，还可用呼吸系统生活质量问卷，如：慢性呼吸系统问卷（Chronic Respiratory Questionnaire, CRQ）和圣乔治呼吸疾病问卷（St. George's Respiratory Disease Questionnaire, SGRQ）等；②生活质量评分：如肺癌治疗功能评价量表（functional assessment of cancer therapy-lung, FACT-L）、欧洲癌症研究和治疗组织（European Organization for Research and Treatment of Cancer, EORTC）的生活质量核心量表（EORTC QLQ-C30）和肺癌的特异量表（EORTC QLQ-LC13）等^[9]^。

## 2 术前肺康复治疗对肺癌手术患者的作用

肺癌患者合并慢性阻塞性肺疾病不仅使肺癌手术术后并发症发生率增加，而且使很多患者失去手术机会^[3]^。由于肺康复治疗对于不手术的COPD患者能明显增加活动耐量和改善生活质量，Wilson^[13]^在1997年提出术前短期、高强度的肺康复治疗可能会减少肺癌手术的并发症和改善预后。Jones等^[14]^首先研究了术前系统的运动训练对20例肺癌手术患者术前和术后心肺功能的影响。患者术前接受为期4周-12周、每周5次强度为60%-100%基线峰耗氧量（baseline peak oxygen consumption, VO_2peak_）的耐力功率自行车训练，在运动训练前、手术前和术后30天均进行CPET、6MWD和肺功能检测（pulmonary function testing, PFT）。结果证实4周-6周的运动训练后VO_2peak_和6MWD都有改善，术前比基线水平相比VO_2peak_增加了2.4 mL/kg/min（15%），6MWD增加了40 m（9%）；而完成80%及以上运动训练的患者改善更加明显，VO_2peak_增加了3.3 mL/kg/min（21%），6MWD增加了49 m（13%）。而肺功能检测并未发现有所改善。这些术前的运动耐量的改善在术后30天有所下降，但均未低于基线值。而且4周-6周与更长时间（8周-12周）的肺康复训练带来的运动耐量改善幅度相似，提示对于某些患者1个月的运动训练就能达到效果。Bobbio等^[15]^的前瞻性研究也报道了类似的结果，其研究入组了12例合并COPD需行手术治疗的临床Ⅰ期或Ⅱ期非小细胞肺癌患者，术前心肺运动试验最大耗氧量（VO_2peak_）≤15 mL/kg/min，经过4周的肺康复治疗，静息肺功能测试和肺弥散功能没有变化，但VO_2peak_平均增加了2.8 mL/kg/min。这两项研究均表明术前的肺康复治疗能改善患者的心肺功能和运动耐量，但是并未证实这些改善是否能够增加患者的手术机会、减少手术并发症和改善患者预后。

为此Cesario等^[16]^研究入组了8例肺癌患者，尽管患者的肿瘤可以切除，但是因患者的身体状况和肺功能而不能手术。这些患者接受了4周的高强度（80%的最大耗氧量）的有氧运动、呼吸训练和健康教育。经过肺康复治疗，患者的6MWD增加了47.4%，PaO_2_增加了7.2 mmHg。与其它研究不同，该研究中患者的肺功能（FEV1和FVC）都有明显改善，而且基线肺功能和运动耐量最差的患者改善最大。这些患者经过肺康复治疗后达到了手术标准并接受了肺叶切除术，术后2例患者出现一过性的并发症（1例出血，1例房颤），但没有患者死亡。

尽管这些研究提示肺癌术前肺康复治疗能改善患者的心肺功能和运动耐量，降低手术并发症，但是目前尚无完善的随机临床试验进行证实，缺乏足够证据推荐肺癌患者术前常规进行肺康复治疗。因此，Benzo等^[17]^设计了两项肺康复治疗的随机单盲临床试验，入选标准是接受肺切除术的肺癌患者，需合并中重度COPD，研究终点是住院时间和术后并发症发生率。第一项研究比较按COPD肺康复治疗指南进行4周的肺康复治疗与常规治疗。但是该研究没有招募到足够的患者，因为绝大部分患者和医生不愿意拖延手术时间，担心4周的治疗耽误手术时机，造成病情进展。最终入组了9例患者，两组结果比较并无明显差异。第二项研究比较术前10次（每天2次）的个体化肺康复治疗方案（包括基于主观评价效果的运动训练、呼吸肌训练和慢呼吸练习）与常规治疗，共入组19例患者。结果显示10次的术前肺康复治疗可以促进术后肺复张，减少胸管放置天数和住院时间，从而减少术后并发症和住院费用。该研究证实术前短期肺康复治疗对于合并中重度COPD的肺癌患者接受肺切除治疗是可行的，但常规的4周治疗方案似乎不可行。

综上，术前肺康复治疗对减少手术并发症、改善预后有积极作用，但需要更多临床研究以探索和规范其方案。对于大部分身体适合手术的患者，往往担心延迟手术可能造成肿瘤进展甚至错过最佳手术时机，限制了肺康复治疗的进行。较为可行的方式是在不推迟手术的情况下，利用术前检查和术前准备的时间，进行短期肺康复治疗。而对于术前肺功能差的患者，立即手术的风险更大，更有时间和必要进行肺康复治疗。美国M.D.Anderson癌症中心对于边缘肺功能或身体状况差的肺癌手术患者的处理方案是：基线评估结果异常的患者均接受综合的肺康复治疗，方案根据基线评估结果（包括肺功能、肺通气灌注扫描、6MWD和心肺运动试验）制定。肺康复治疗方案一般包括每周3次高强度（60%-80%的VO_2peak_）的运动训练，共3周-6周，治疗结束后重新评估肺功能（心肺运动试验和6MWD）有明显提高的患者再考虑手术。运动训练没有严格的目标，但一般VO_2peak_达到60%以上、6MWD > 400 m方可考虑手术，最后由外科医师决定是否手术。肺康复治疗在术后继续进行6周-9周^[18]^。

## 3 术后肺康复治疗对肺癌手术患者的作用

肺癌术后由于肺组织容量减少，加上手术创伤，必然导致术后心肺功能损害、呼吸生理紊乱、运动耐量下降、活动受限、表现呼吸困难等症状，肺康复治疗在这些方面是否会发挥积极作用？Spruit等^[19]^研究了术后8周的肺康复治疗对于功能运动耐量和峰运动耐量的作用。10例接受了肺切除术的肺癌患者在术后平均3个月开始接受肺康复治疗，基线肺功能检查显示均有中到重度的COPD，治疗前后均评价了患者的肺功能、6MWD和峰运动耐量变化，肺康复治疗后功能运动耐量（43.2%）和峰运动耐量（34.4%）都有明显提高，尽管肺功能没有明显改善。基于这些初步结果，作者提出肺癌接受抗肿瘤治疗后均适合并且有明确指征开始综合的肺康复治疗。Cesario等^[20]^报道了25例肺癌患者术后开始进行4周的肺康复治疗的效果，2001年-2004年住院接受肺切除术患者中211例符合入组标准，其中25例患者接受了肺康复治疗，其余186例患者拒绝进行肺康复治疗而作为对照，研究评价了治疗前后的6MWD、Borg呼吸困难评分、血气及肺功能变化。结果显示接受肺康复治疗的患者6MWD和Borg呼吸困难评分都有提高，相反，对照组患者这些指标在术后均有下降。而且尽管基线水平治疗组明显差于对照组，治疗后两组比较无明显差异，提示术后4周的肺康复治疗改善了肺切除患者的运动耐量和呼吸困难程度，并提出肺康复治疗可作为肺癌手术综合治疗的一部分，并且不影响肺癌术后其它的辅助治疗。Varela^[21]^进一步对围手术期肺康复治疗进行了经济学方面的分析，研究中119例肺癌患者从术前第1天开始并在整个住院期间进行运动和呼吸训练。术后患者的肺不张发生率和住院天数明显低于同期520例对照患者，这些节约的费用已足够抵消肺康复治疗的相关费用。

通过上述研究可以发现术后肺康复治疗对于心肺功能的恢复和呼吸困难等症状改善的作用，而规范的术后肺康复治疗需要更有力的临床试验证据支持，目前已经有相关的随机临床研究开展。肺癌运动训练研究（LUNGEVITY）^[22]^是一项正在进行的随机临床试验，目的是研究不同的运动训练方式对于肺癌患者术后心肺功能的影响，以峰耗氧量（VO_2peak_）为评价指标，以期找到肺癌患者最佳的运动训练方式并探讨其机制。该临床试验计划入组160例经过根治性手术切除并且病理证实的Ⅰ期-Ⅲa期肺癌患者，符合入组标准的患者被随机分为4个治疗组：①有氧运动组；②耐力训练组；③有氧运动和耐力训练组；④对照组（伸展运动）。各组运动训练的目标是为期16周、每周3次、每次30 min-45 min的运动训练，有氧运动强度在70%的最大耗氧量以上，耐力训练强度为60%-80%的最大肌力。首要研究终点是VO_2peak_，次要终点包括患者报告结果（patient-reported outcomes, PROs）（如生活质量、疲乏、抑郁等）和“氧级联”相关器官的功能（如肺功能、心功能、骨骼肌功能等），运动训练前后对所有研究终点进行评估。亚组研究包括个体对运动刺激的基因研究、运动依赖的理论决定因素、运动-PRO的心理调节因素、运动导致的基因表达变化。

## 4 肺康复治疗对肺癌非手术患者的作用

绝大部分肺癌患者确诊时往往已到晚期，加上部分因身体情况而不能手术的患者，其治疗手段主要是放化疗和靶向治疗。肺癌患者通常由于肿瘤本身会有乏力和呼吸困难等症状，加上各种治疗措施，特别是放化疗的副作用，严重影响了肺癌患者的生活质量。对这些患者进行肺康复治疗，可以减轻疲乏、呼吸困难等症状，并缓解放化疗的副作用^[10, 23]^。Temel^[24]^入组了25例晚期肺癌患者进行8周的耐力和力量训练，肺康复训练同时进行化疗。结果显示仅44%的患者完成了8周的训练，而完成训练的患者运动耐力增加，且疲乏等症状减轻。Glattki^[25]^研究了47例肺癌患者抗肿瘤治疗之后进行综合的肺康复治疗对于肺功能和运动耐量的作用，其中包括18例晚期非手术患者。在治疗前后检测了入组的47例患者的肺功能、动脉血气、6 min步行测试和呼吸困难程度。结果患者的1秒钟用力呼气容积（forced expiratory volume in first second, FEV1）[平均增加（110±240）mL；*P*=0.007]、用力肺活量[平均增加（130±290）mL；*P*=0.001]、6 min步行距离（平均增加41 m；*P* < 0.001）都有明显提高。呼吸困难明显改善，MMRC呼吸困难评分平均降低0.26±0.61（*P*=0.007）。该研究证实肺癌患者在接受了抗肿瘤综合治疗后进行肺康复治疗，能够改善肺功能和运动耐量，且改善效果与是否合并COPD及是否手术治疗无关。

然而我们也看到，对非手术的晚期肺癌患者进行康复治疗很有挑战性，大部分患者身体状况不允许进行肺康复治疗，甚至无法进行心肺运动试验检查，而参与治疗的患者过半未能坚持完成治疗。对于这部分患者的肺康复治疗，需要更加个体化的方案，其安全性和可行性还需要更多的临床研究论证。

## 5 问题与展望

尽管肺康复治疗在慢性肺阻塞性疾病治疗中得到广泛重视和应用，其提高患者运动耐力、减轻呼吸困难症状和改善生活质量的疗效也得到大量临床资料的证实和认可。肺康复治疗在肺癌中的应用还处于探索阶段，但初步的临床研究已显示它能有效地改善肺癌患者的运动耐量和生活质量、减少手术并发症并增加手术机会，具有重要的应用前景。尽管在我们临床工作中有一定应用，包括鼓励患者进行吹气球、咳嗽训练以及术前进行爬楼梯等锻炼，但是并未得到足够重视和规范，国内尚无相关临床研究，国外也是近来来才开始有为数不多的小样本临床研究，主要是对运动训练作用方面的观察研究，缺乏对肺癌患者心肺功能损害机制的研究以及肺康复治疗的机制研究。肺康复治疗的方案多是参照COPD患者治疗的方案，对于术前术后运动训练的时间、强度和频率等缺乏适合肺癌患者的规范。相信随着越来越多临床研究的开展，肺癌患者的肺康复治疗将得到进一步的重视和规范，并将成为肺癌综合治疗的重要组成部分，以进一步提高肺癌的治疗水平并改善肺癌患者的生活质量。
